# LongISLND: *in silico* sequencing of lengthy and noisy datatypes

**DOI:** 10.1093/bioinformatics/btw602

**Published:** 2016-09-25

**Authors:** Bayo Lau, Marghoob Mohiyuddin, John C. Mu, Li Tai Fang, Narges Bani Asadi, Carolina Dallett, Hugo Y. K. Lam

**Affiliations:** 1 Roche Sequencing Solutions, Belmont, CA 94002, USA; 2 Roche Sequencing Solutions, Pleasanton, CA 94588, USA

## Abstract

**Summary:** LongISLND is a software package designed to simulate sequencing data according to the characteristics of third generation, single-molecule sequencing technologies. The general software architecture is easily extendable, as demonstrated by the emulation of Pacific Biosciences (PacBio) multi-pass sequencing with P5 and P6 chemistries, producing data in FASTQ, H5, and the latest PacBio BAM format. We demonstrate its utility by downstream processing with consensus building and variant calling.

**Availability and Implementation:** LongISLND is implemented in Java and available at http://bioinform.github.io/longislnd

**Contact:** hugo.lam@roche.com

**Supplementary information:** Supplementary data are available at *Bioinformatics* online.

## 1 Introduction

LongISLND is a software package designed to emulate third-generation single-molecule sequencing (SMS) technologies (Eid *et al.*, 2009; Jain *et al.*, 2015; Kumar *et al.*, 2012; Ventra, 2013). Development, deployment and adoption of new sequencing technologies can be accelerated by proof-of-concept downstream analyses using simulated data, avoiding expensive and laborious experiments. Such analyses, for example, include alignment (BLASR/GraphMap), error correction and variant calling (Quiver/PBHoney/Nanopolish) and genome assembly (MHAP/Falcon/MiniASM) (Berlin *et al.*, 2015; Chaisson and Tesler, 2012; Chin *et al.*, 2013; English *et al.*, 2014; Li, 2016; Loman *et al.*, 2015; Pendleton *et al.*, 2015; Sovic *et al.*, 2016). Development of SMS can be further accelerated by testing with realistic simulation of various chemistries and use cases.

To date, simulation has been hampered by the lack of a realistic yet versatile long-read simulator. For example, PBSIM (Ono *et al.*, 2012) generates only the FASTQ data format, without the multi-pass mechanism or additional per-base probability and kinetic data required by downstream analysis tools. The Alchemy simulator in the BLASR package (Chaisson and Tesler, 2012) is unmaintained and generates data for an older PacBio format incompatible with modern downstream analysis tools. In addition to practical usability issues, a general concern is that the simulators are tailored to a particular chemistry of a particular sequencing technology, resulting in idealized error models which might not capture true error characteristics.

These issues have been addressed by our new software package LongISLND. The method is designed to be independent of the underlying sequencing mechanism. The implementation is polymorphic with respect to output file formats. We train our software for multiple PacBio chemistries, instantiate output for multiple file formats, then demonstrate its utility by VarSim (Mu *et al.*, 2014) evaluation of PacBio’s latest CCS2 consensus builder (https://github.com/PacificBiosciences/pbccs) and of various germline and somatic variant callers for PacBio reads.

## 2 Methods

A sequencing experiment generates multiple reads, each a series of basecalls based on an optimally tuned combination of chemistry, sequencing and primary analysis. Bioinformaticians pay most attention to the quality of the resulting basecalls. To remain platform-agnostic, LongISLND uses a learn-and-simulate approach detailed in the Supplementary Material. Briefly, real data is aligned to truth sequences. The alignment records are then analyzed to extract a non-parametric model, from which error profile can be extracted and according to which simulation is performed for a set of target sequences, such as a genome (Supplementary Fig. S1). The learning step records samples for a given set of real data, forming an empirical model. The software architecture allows for sampling highly customizable features; for instance, data in the popular FASTQ format may sample basecalls and quality values (QV), but additional features may be sampled as well. For example, with PacBio data the architecture supports the inclusion of the Phred probability of a base being an insertion (*Q*_ins_), the Phred probability of the previous base having been deleted (*Q*_del_), the most probable deleted base (*tag*_del_), the Phred probability of the last base having been combined with current base (*Q*_merge_), the Phred probability of the current base being a substitution (*Q*_sub_), the most probable substituted base (*tag*_sub_), the overall Phred quality value (QualityValue) and the separation between two signal pulses (IDP). To the best of our knowledge, PacBio’s QualityValue feature correlates with the probability of no ins/del/sub/merge and is used as the QV in the FASTQ format.

**Fig. 1.**
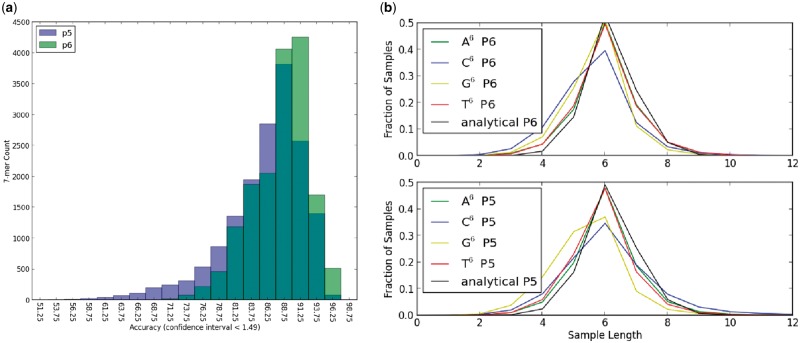
(**a**) Number of 7-mers binned with respect to accuracy, determined within 1% as discussed in the Supplementary material. A context-independent error profile would yield a delta peaking function centered at the global accuracy. (**b**) Fraction of samples of a certain sequence length aligned to a homopolymer of true length 6. Compared to the analytical expression derived in the Supplementary Material, G/C deletion bias is observed in both P5 and P6 chemistries

LongISLND learns from alignment data by recording base calls, with and without error, according to sequencing contexts of the reference. Single molecule sequencing, either by synthesis (Eid *et al.*, 2009; Kumar *et al.*, 2012) or by direct measurement (Ventra, 2013), probes the nucleotide sequence incrementally. Such technologies aim to achieve position-independent error rates; however, the error rate can be sensitive to the short-range sequencing context which may affect a range of characteristics from steric and chemistry properties to analog signal processing (Eid *et al.*, 2009; Kumar *et al.*, 2012; Ventra, 2013). Another source of error is the inference of homopolymer count from analog signal. Such inference can have homopolymer-length-dependent bias (see Supplementary Material) and, in the case of insertion-deletion cancellation, the inserted bases might not be the same as the deleted bases. The relevant sequencing context can be categorized by the length and base identity of the homopolymer, as well as the identity of flanking bases. We call this information an extended-k-mer (EKmer), detailed in the Supplementary Material. In brief, the use of EKmer facilitates analysis and simulation based on empirical sequencing context without assuming an analytical model. Ordinary k-mer-based analysis is limited to contexts shorter than or equal to k base-pairs; in particular, k-mer-based methods would collapse all information of all homopolymers of length *L *>* k* to that of length *k*. EKmer can be applied to homopolymer whose length exceeds *k*. The importance of capturing and replicating homopolymer error profiles has been demonstrated in the literature (Ross *et al.*, 2013), the Supplementary Material and Figure 1b.

Simulation is performed as follows: according to the fragment distribution of the empirical model, LongISLND draws a simulation fragment from a set of user-specified FASTA genomic sequences. Table 1 illustrates the processing of a simulation fragment. The fragment is treated as the truth and translated into a series of EKmer contexts. For each context in the series, an event type (insertion/deletion/substitution/match) is randomly drawn according to the context-specific frequency recorded in the empirical model. From an EKmer- and event-type-specific bin, a set of basecalls and per-base data are randomly drawn and appended to an output buffer. For example, a match event would append matching basecalls and a deletion event would append a shortened homopolymer (possibly of length zero). The simulated read sequence is assembled from the buffer when the whole series has been processed.

**Table 1.** Step N to N + 5 of a series of extended-k-mer operations across a hypothetical truth sequence

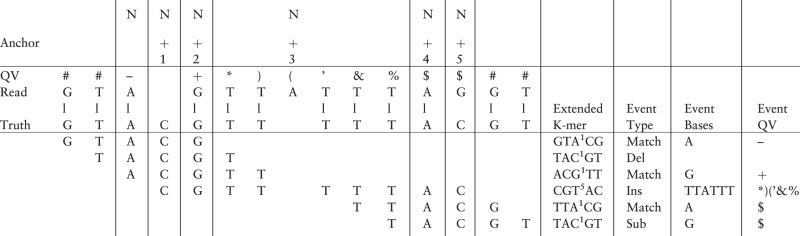																			

Number of flanking bases is taken to be a small value of 2 for illustration only. At step *N*, the truth has an A flanked by GT and CG, this is characterized by GTACG. The read has a matching A, so a Match event is recorded/simulated. At step *N* + 1, the truth has a C flanked by TA and GT, this is characterized by TACGT. The read has a deleted C, so a Deletion event is recorded/simulated. At step *N* + 3, the truth is a length-5 T homopolymer flanked by CG and AC, this is characterized by CGT^5^AC. Over the stretch of homopolymer, one A is inserted in the read, and a insertion event is recorded/simulated.

The empirical model can also provide optionally the number of passes, *N*_p_, which is unity for single-pass sequencing and greater than 1 for multipass sequencing. The above context-series processing is repeated for each of the *N*_p_ passes, alternating between the forward and reverse strands of the simulation DNA fragment. The results are concatenated with adapter sequences to form a full read, which is then fed into an output generator instance. For PacBio reads, we have implemented generators for FASTQ, H5 and the new PacBio BAM formats.

One potential use of a read simulator is to test a hypothetical error profile. We allow such usage by accepting custom rates of insertion, deletion and substitution. For each EKmer considered in the simulation, we first randomly draw an event type according to the custom rates, then randomly draw a corresponding set of basecalls. With the software hooks for modifying error rate, position-dependent errors, such as quality degradation towards sequence ends, can be easily added via position-dependent scaling. We note that the distribution of fragment lengths and numbers of passes are also modifiable. We also note that LongISLND can take into account the variations among different sequencing runs by merging multiple empirical models.

## 3 Results and discussions

The importance of context-dependency was demonstrated by a comparison between PacBio’s CHM1 human datasets generated using P5 and P6 chemistries. Supplementary Table S1 shows that the bulk accuracy increased from 86% to 88% with a P5-to-P6 upgrade; furthermore, sequencing error non-uniformity had been significantly reduced as shown in Figure 1a. For example with P5 chemistry, a 0.15 fraction of sequencing context had <80% accuracy. Such fraction was decreased to 0.05 for P6. Figure 1b shows the homopolymer bias as captured by EKmer. Supplementary Figure S2 shows that the homopolymer bias, with respect to both length and base composition, also changed significantly enough that bioinformatic tools should treat P5 and P6 data with different models. Capturing these biases is one improvement over existing long-read simulators (Chaisson and Tesler, 2012; Ono *et al.*, 2012).

Another valuable and unique feature of LongISLND is the easily customizable output format, which is crucial in facilitating apples-to-apples comparisons among a wide range of tools. Supplementary Figure S4 demonstrates FASTQ, PacBio H5 and PacBio BAM output, with empirical models of PacBio P5 and P6 chemistry. Supplementary Tables S2 and S3 establish the realism of LongISLND by demonstrating model convergence and by comparing the coverage-dependent variant calling accuracy as computed by PacBio’s BLASR + QUIVER pipeline using real and simulated *E.coli* data. Supplementary Table S5 further demonstrates agreement in variant calling results using real NA12878 data (Pendleton *et al.*, 2015) and simulated reads. We demonstrated downstream processing of 10-to15-pass of high-error circular sequenced reads (Eid *et al.*, 2009) with PacBio’s latest CCS2 consensus builder, whose output is evaluated to have >99% mappability and Q30 median accuracy. We also demonstrated the VarSim (Mu *et al.*, 2014) evaluation of several off-the-shelf variant calling pipelines, some of which require PacBio-specific data. Supplementary Table S5 also demonstrates significant difficulties in detecting heterozygous variants with P5C3 chemistry. We found that FreeBayes (Garrison and Marth, 2012) yielded the best diploid SNV accuracy and Quiver (Chin *et al.*, 2013) yielded the best diploid Ins/Del accuracy. Furthermore, we observed significant increase in SNV accuracy at 200X. Supplementary Table S6 shows that VarDict (Lai *et al.*, 2016) yielded the best somatic mutation accuracy.

Although a large Oxford Nanopore dataset is thus far unavailable to us for detailed analysis, the use of LongISNLD and GraphMap to the learn and simulate according to a small Oxford Nanopore R7.3 dataset (Loman *et al.*, 2015) is demonstrated in the Supplementary Material.

LongISLND currently captures context-dependent yet positionally uniform errors found in single-molecule sequencing technology. Error features longer than the EKmer length have not been captured. One long-range error feature is random patches of garbage data. In the context of the PacBio H5 convention, usable reads are separated from garbage reads by a high-quality-read-region flag. If garbage stretches of basecalls are inadvertently included in the high-quality region, the empirical long reads could contain long patches of garbage data (https://dazzlerblog.wordpress.com/2015/11/06/intrinsic-quality-values). Another long-range error feature is chimeric reads. Emulation of such structural errors would require alignment junction analyses.

## 4 Conclusions

LongISLND is an accurate simulator that uses context-dependent error profiles to realistically simulate single-molecule sequencing technologies. It is a valuable tool for bioinformaticians who want to tune for bias in sequencing characteristics, as well as for primary-analysis developers who want to predict the performance of downstream analyses. The simulator can be easily extended to support various I/O formats required by different bioinformatic tools.

## Supplementary Material

Supplementary Data
