# Supplementary material for: LongISLND: in silico sequencing of lengthy and noisy datatypes
Source: Bioinformatics. 2016 Sep 25;32(24):3829–32. doi: 10.1093/bioinformatics/btw602 (PMC5167071; doi:10.1093/bioinformatics/btw602)
Supplement: Supplementary Data [file supp_32_24_3829__index.html]

LongISLND: in silico sequencing of lengthy and noisy datatypes — LongISLND: in silico sequencing of lengthy and noisy datatypes — Supplementary Data 

# LongISLND: *in silico* sequencing of lengthy and noisy datatypes

## Supplementary Data

files

- Supplementary Data - pdf file
