# Supplementary material for: LongISLND: in silico sequencing of lengthy and noisy datatypes
Source: Bioinformatics. 2016 Sep 25;32(24):3829–32. doi: 10.1093/bioinformatics/btw602 (PMC5167071; doi:10.1093/bioinformatics/btw602)
Supplement: Supplementary Data [file supp_btw602_LongISLND_Supplementary-20160817.pdf]

SUPPLEMENTARY MATERIALS

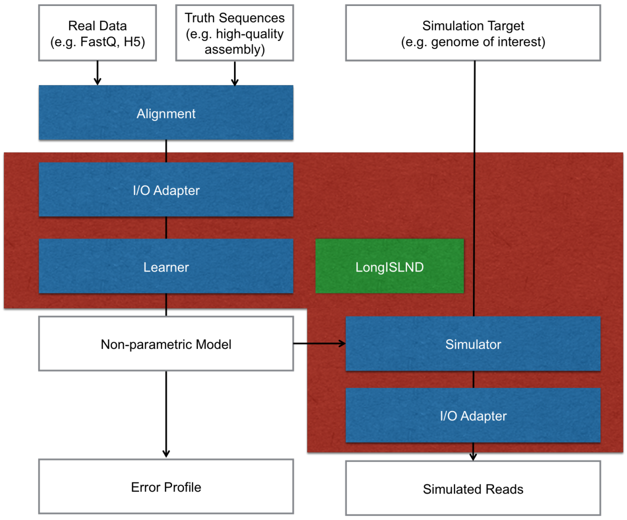

FIGURE S 1. Architecture of the LongISLND simulator. Real data is aligned to truth sequences, such as a high-quality de-novo assembly. The alignment records are then analyzed to extract a non-parametric model, from which error profile can be extracted and according to which simulation is performed for a set of target sequences, such as a genome.

1. EXTENDED K-MER

For each base at locus i of the reference genome, we define a k-mer function,

$$\hat{K}_{lr}(i),$$

which returns a $(1+l+r)$-mer of the bases from $i-l$ to $i+r$. We use the variable K_{lr} to denote kmer values returned by $\hat{K}_{lr}(i)$. Next, we define a homopolymer-compressed k-mer function,

$$\hat{e}_{LR}(i),$$

which returns a $(1+L+R)$-mer of the base at i, L bases prior to the homopolymer covering i, and R bases after the homopolymer covering i. We use the variable e_{LR} to denote values returned by $\hat{e}_{LR}(i)$. We also define $\hat{H}(i)$ which returns the integer length of the homopolymer covering i. Formally speaking, an extended-k-mer (EKmer) can be derived using the composite function

$$\hat{E}_{LR}(i) = \left(\hat{e}_{LR}(i), \hat{H}(i)\right).$$

We use the variable E_{LR} to denote EKmer values returned by $\hat{E}_{LR}(i)$. For example, at anchor position N in Table 1, $\hat{E}_{LR}(i)$ would return E_{22}=(GTACG, 1). At all five T positions within anchor position $N+3$ in Table 1, the function would return E_{22}=(CGTAC, 5).

As discussed in Method section, expressing the truth sequence as a series of E_{LR} captures possible sequencing-context dependencies due to the incremental, single-strand nature of nucleotide measurement, and due to the difficulties that may occur in inferring homopolymer count from the analog signal. We point out that allowing $L \neq R$ provides platform-specific tuning if the sequencing characteristic of a particular platform is more dependent to either side of the physical nucleotide probe. We note that, for example, the sole use of K_{22} [2] can reproduce short-range context-dependent sequencing characteristics; however, if the homopolymer bias is length-dependent (as shown below in Error Profile section), the limited range of K_{22}

	Ins (%)	Del (%)	Sub (%)	Accuracy (%)
P5	7.0	5.4	1.2	86.4
P6	6.3	4.4	0.9	88.3

TABLE S 1. Bulk error rate of P5 and P6 chemistries.

size would induce aliasing effects, essentially treating all homopolymers with identical error rate. Lastly, we note that reverse-complemented k-mers can be distinguished from those in the forward orientation. Single-molecule sequencing can be strand-specific, and the reads and alignment data can preserve strand-specific information [9, 6], allowing strand-specific analysis of sequencing bias. Distinguishing reverse-complemented k-mers allows LongISLND to implement a strand-aware learn-and-simulate mechanism.

2. Learning

LongISLND constructs an empirical model as a collection of key-value pairs for each base at locus i of the reference genome. The key can take on values of all K_{lr} and E_{LR}. The value of a key-value pair is an event. An event consist of a type (insertion/deletion/substitution/match) along with the data specific to the sequencing technology. For example, basecalls and QVs are stored for ordinary FASTQ data, whereas basecalls, QV, Q_{ins}, Q_{del}, Q_{merge}, Q_{sub}, tag_{del}, tag_{sub}, and IDP [9, 6] are stored for PacBio data.

We take an empirical measurement approach to be platform-agnostic. Reads are first aligned to a reference. For example, the human hydatidiform mole cell line CHM1hTERT has been sequenced with PacBio's P5 [3] and P6 chemistries [4] and can be aligned to the MHAP assembly [1].

LongISLND iterates over the alignment records. For each reference base i, $\hat{K}_{lr}(i)$ is used to obtain a K_{lr}, which is associated with an event containing the read sequence aligned to the base at i. If i marks the beginning of a homopolymer, $\hat{E}_{LR}(i)$ is also used to obtain an E_{LR}, associated with an extra event containing the read sequence aligned to the all $\hat{H}(i)$ bases of the homopolymer. The data representations provided by modern sequencers provide additional error metrics beyond those of simple FASTQ. For example, extra per-base meta data [9, 6] can facilitate higher accuracy of downstream analysis. LongISLND's polymorphism allows association of such meta data into an event. LongISLND samples all events from all alignment records. To improve I/O performance, LongISLND allows down-sampling of the collected events before streaming the events into storage. For example, the user can specify that a maximum of 100 samples per key per event-type (ins/del/sub/match) be collected.

Depending on alignment parameters, read-to-reference aligners tend to group indels together based on the scoring scheme and left-alignment criteria. This is a potential problem for high-indel-error technologies. We shift the aligned bases around to make sure indels don't clump together, constrained by the number of matches being equal.

Sequencing technologies have their own physical characteristics. For example, the PacBio sequencer operates with the multi-pass mechanism. For a given DNA fragment, the readout has a rough pattern of forward-strand, adaptor, reverse-strand, adaptor, repeating until termination. The full readout is called a polymerase read, and the strand readouts (separated by the adaptors) are sub-reads. For PacBio reads, LongISLND collects such patterns from the reads. Each collected sample contains a set of sub-read lengths. The DNA fragment size is approximated from the sub-read distribution, and the number of forward-reverse cycles is approximated from the number and length of sub-reads.

The realism of the model increases with increasing the number of flanking bases; however, the maximum flanking base-pairs is limited by available alignment data as well as the sequencing context present in a reference. We found that the CHM1 data sets have enough data to sufficiently populate all events of all K_{44} and E_{22} keys; however, due to the amount of data as well as genome sequence contexts, the E. coli data set [7] can only sufficiently populate all events of all keys of K_{22} and E_{22}.

3. Error Profile

The utility of LongISLND becomes apparent as its application immediately reveals differences in the data properties derived from PacBio's P5 and P6 chemistries. We used BLASR [2] to align the publicly available CHM1hTERT datasets [3, 4] to the MHAP assembly [1], and then extracted the error profile. Table S1

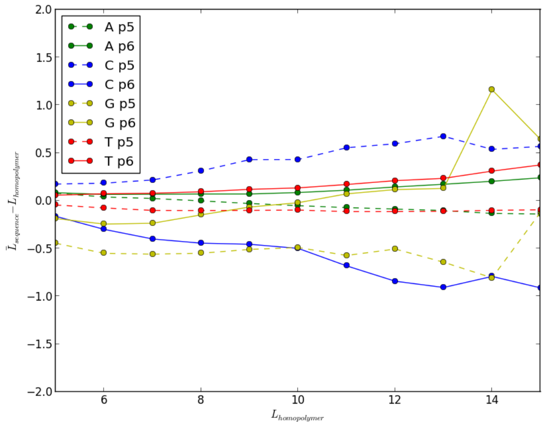

FIGURE S 2. Average readout length minus the true homopolymer length.

shows noticeable P5-to-P6 improvement in bulk error statistics, obtained by summing ins/del/sub/match events over all K_{44} keys.

Moreover, our context-dependent sampling reveals significant reduction in non-uniformity. We first consider the accuracy of the conventional 7-mer contexts, which is merely K_{33}. For each K_{33}, we count $N_{K_{33}}$, the number of 7-mer appearances in the reference sequences of alignment records, as well as $M_{K_{33}}$, the number of those appearances without sequencing error. $M_{K_{33}}/N_{K_{33}}$ is a ratio between 0 and 1, and is an estimator for the accuracy of sequencing a K_{33} context. We have processed enough sample data to determine the accuracy of all K_{33} contexts within 1%; in particular, the maximum range of $\alpha = 0.05$ confidence interval is less than 1.49% (narrower than then bin width of Figure 1) as determined by the script `paper/estimate_accuracy.py` in the LongISLND package. Figure 1 in the main text displays the number of K_{33} sequencing context as a function of accuracy. Since accuracy is determined within 1%, the plot clearly demonstrates significant improvement in the context-uniformity of error profile. For example, we see that the fraction of k-mer context with accuracy lower than 80% improves from ~ 0.15 for P5 chemistry to ~ 0.05 for P6 chemistry. The P5-to-P6 improvement is more than just a mere 2% increase in overall accuracy. It also includes a significant decrease in the number of sequencing contexts with low error rate.

Next, we demonstrate the emulation of homopolymer bias. For a context-independent error profile, the probability of observing a sequence length L', for a given true length L, is dependent on the bulk insertion and deletion probabilities, p_{ins} and p_{del}, respectively. As a first-order approximation, one can treat the basecalling process as L independent events of single-base insertion (I), single-base deletion (D), or no indel (N). The number of basecalls generated by a particular set of events is $2I + N$. Since $L = I + D + N$, the probability of sequencing L' given L can be approximated as

$$P(L'|L) = \sum_{\substack{I,D,N>=0 \\ I+D+N=L}} \frac{L!}{I!D!N!} p_{ins}^I p_{del}^D \left(1 - p_{ins} - p_{del}\right)^N \delta\left(L' - 2I - N\right),$$

where the delta function returns one if $L' = 2I + N$ and zero otherwise. p_{ins} and p_{del} are estimated by the number of bases inserted and deleted, respectively, relative to the number of reference bases in the alignment. Figure 1b in the main text compares the sampled homopolymer readout against the analytical expression. LongISLND captures subtle yet critical characteristics. For example, we see the drastic improvement of G-deletion bias, from P5-to-P6 chemistry.

We further calculate, for each homopolymer length, the average readout length minus the homopolymer length. This quantity should be 0 without bias, and an average of +/-0.5 could be interpreted as heterozygous variant by a naive pile-up variant calling method. Figure S2 shows improvement from P5 to P6 chemistry, but C/G indel bias is noticeable even with P6 chemistry.

4. Simulation Details

LongISLND's simulation procedure has been explained in the main text. Here, we point out additional details about the implementation.

Consider the CHM1 data set which populates all K_{44} and E_{22} keys. During simulation, we choose our simulation EKmer to be E_{44}. For a given simulation fragment, LongISLND processes each E_{44} as follows. If $H = 1$, we reinterpret E_{44} as the equivalent K_{44}, and an event is drawn from the pool of K_{44}. If $H \neq 1$, we reinterpret E_{44} as E_{22} by discarding the extra flanking bases. If the homopolymer lengths of all events in E_{22}'s bin has enough samples, we draw an event from the bin. Otherwise, for example if a 1000bp homopolymer never existed in the training data, we decompose the sequence captured by E_{44} as a series of K_{44}, then process a random event for each K_{44}. This is merely a fail-safe way of applying the EKmer method with limited data.

We note that LongISLND can take into account the variations among different sequencing runs. This is supported by the capability of merging the fragment distribution as well as key-value pairs of multiple empirical models. In this scenario, the E_{LR}-dependent error rate is averaged over all models, and the actual sequencing events are drawn uniformly out of all models.

5. Realism of Simulation

The impact of the number of flanking bases can be illustrated by comparing the accuracy of K_{ff} and the sixteen $K_{f+1,f+1}$'s obtained by sandwiching A/C/G/T. For each of the 4^{2f+1} K_{ff}, we perform a simple chi-squared categorical test for a 16×2 table: each cell contains the count of A/C/G/T-sandwiched K_{ff} with or without a sequencing error. The null hypothesis is that the accuracy is independent over the 16 $K_{f+1,f+1}$'s formed with an extra flanking base pairs on both ends. Table S2 shows that the fraction of K_{ff} with $\alpha = 0.001$ rejected null hypothesis. The fraction decreases with increasing f. A significant decrease of rejection is observed going from $f = 3$ to $f = 4$, signifying a drastic increase of independent $K_{f+1,f+1}$.

flank (f)	Fraction of K_{ff} with accuracy independent of extra flanking base pairs
4	0.14
3	0.74
2	1.0

Table S 2. The fraction of K_{ff} with $\alpha = 0.001$ rejected null hypothesis that the accuracy is independent for the 16 $K_{f+1,f+1}$'s derived by extra flanking base pairs. Calculation is performed for P6 CHM1 data.

We further evaluate variant-calling accuracy using real and simulated E. coli data of P6 chemistry. Real P6 data, R, and the high-quality assembly, G, are downloaded (https://github.com/PacificBiosciences/DevNet/wiki/E.-coli-Bacterial-Assembly). We use LongISLND to learn from the alignment of R against G and to generate simulated reads, S, in the PacBio H5 format. A new reference, G', is generated by randomly modifying G with random mutations. We use BLASR to align R and S against G', Quiver to call variants against G', and VarSim to evaluate accuracy. Quiver was invoked with P6-specific setting, "-x 5 -q 20 -pP6-C4.AllQVsMergingByChannelModel", to take full advantage of PacBio non-standard QVs and chemistry tuning. Table S3 shows good agreement between the F1 accuracy score between the real data and LongISLND simulated data.

Coverage	Type	Real P6	$f = 4$	$f = 2$
20	SNV	1.000	0.999	0.998
20	INS	0.912	0.925	0.930
20	DEL	0.991	0.984	0.987
30	SNV	1.000	1.00	0.999
30	INS	0.984	0.984	0.993
30	DEL	0.997	0.993	0.996

Table S 3. Comparison of variant calling accuracy between real and simulated data of E. coli.

6. Generalized Implementation

LongISLND's architecture is shown in Figure S1. LongISLND has been implemented generically using Java Interfaces and Abstract Classes, allowing the simulator to be extended to cover different file formats and data sampling strategies.

An alignment record is abstracted as an `EventGroup`. An alignment file is abstracted as `Iterable<EventGroup>`. The aforementioned learning mechanism is abstracted as an `EventGroupProcessor`, which processes a set of `EventGroup` to construct an empirical model represented by a class `Samples`. The simulation mechanism is implemented to obtain truth sequences from `RandomFragementGenerator`, to generate corresponding simulated sequences according to the empirical model in `Samples`, and to output the simulated sequences using an implementation of `ReadsWriter`.

The actual implementation and functionality of these abstract classes are determined by an `enum Spec`. At run time, a particular value of `Spec` is determined based on user input. Such `enums` specify implementation based on sets of `enum`. Examples of `Spec` values are `PBBaxSpec`, `PBCcsSpec`, `PBClrBamSpec`, and `SAMFastqSpec`, for respectively, bax.h5, ccs.h5, PacBio BAM, and ordinary Fastq/BAM format.

As a concrete example, a user can specify `PBBaxSpec` for the learning step and `PBClrBamSpec` for the simulation step. This choice would tell LongISLND to extract a model from PacBio's h5 format [9], including per-base meta data of basecall, QV, Q_{ins}, Q_{del}, Q_{merge}, Q_{sub}, tag_{del}, tag_{sub}, and IDP. Using the generated model, the simulation step would simulate reads into the PacBio BAM format [6]. This is due to the fact that the choice of `PBClrBamSpec` selects the `BAMWriter` as the implementation of `ReadsWriter`.

This architecture allows LongISLND to be easily extendable with minimal addition of, e.g., new values of `Spec` and child classes of `ReadsWriter`.

7. Installation, Data, and Execution

Please see detailed instructions at https://github.com/bioinform/longislnd. Briefly, a Linux or OSX system with Java 1.7+ and Maven is required. Maven can be obtained easily, e.g., using the automated script in https://github.com/bayolau/build_env. After a GIT clone of https://github.com/bioinform/longislnd, the software can be built by running `linux_build.sh`. The build system automatically pulls in dependencies such as HDF Java and HTSJDK.

Execution is demonstrated in `sample.py` and `simulate.py`. Example usages can be found in the `sampling_example` directory.

8. Application to Oxford Nanopore Data

Although human-scale data of Oxford Nanopore data is thus far unavailable to us for detailed analysis, we demonstrate LongISLND's application to a small set of R7.3 data [5]. First, one should install GraphMap and Samtools by executing setup_ont.sh in the longislnd/sampling_example directory. One should then enter the longislnd/sampling_example/ont_ecoli directory and execute download_and_align.sh to download the E. coli data set [5] and the E. coli reference as well as to align the reads to the reference using LongISLND and GraphMap. The learn_and_simulate.sh in the same directory would learn from the alignment of 2D reads and simulate a FASTQ. Due to the relatively small data set, the confidence interval of K_{33} accuracy as calculated in Sup. Sec 3 is rather large compare to those derived from PacBio CHM1 data. To facilitate a meaningful comparison, we compute, for each K_{33}, the posterior probability, under the binomial proportion model. We use the posterior probability to compute the average number of 7-mer in each accuracy bin as shown in Fig. S3. The figure clearly demonstrated that the 2D reads have an error profile distinct from that of PacBio.

9. Evaluation Framework

Figure S4 shows various evaluation configurations facilitated by VarSim and LongISLND. VarSim generates a realistic simulated genome, which is then randomly fragmented by LongISLND for simulated reads from shotgunned DNA. After processing with the pipeline of choice, evaluation is performed by VarSim which knows the true variant calls. The figure demonstrates the generation of both H5 and PacBio BAM formats as required by different bioinformatic pipelines.

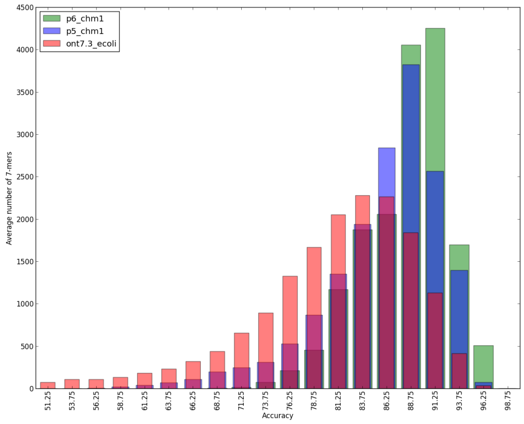

FIGURE S 3. Average number of 7-mers with respect to accuracy, as calculated from posterior probability due to low coverage, as discussed in text.

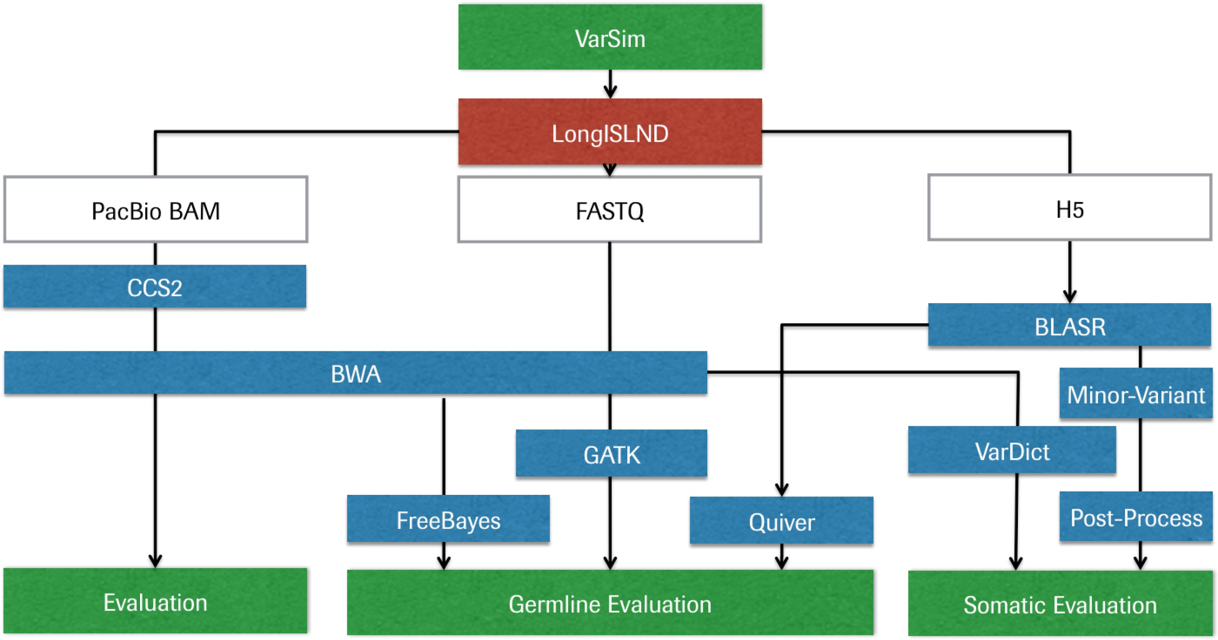

FIGURE S 4. LongISLND generates CCS/CLR reads according to VarSim's simulation. The simulated reads are processed by various workflow before evaluation.

10. CCS2 Evaluation

The build tools in https://github.com/bayolau/build_env enable building of a pre-release version of CCS2 code from the following list of GIT repositories

- https://github.com/PacificBiosciences/pbccs 634f149a167969f07ed58ccf58739c8ddf6fe745
- https://github.com/PacificBiosciences/htslib 6b6c81388e699c0c0cf2d1f7fe59c5da60fb7b9a
- https://github.com/PacificBiosciences/ConsensusCore2 94aa865939f50a6be668e101fe3567cf1ad5e9d1
- https://github.com/PacificBiosciences/pbbam 6be3a1b2711c31772f7f79d292e13b76dd8f9d8b
- https://github.com/PacificBiosciences/seqan a1fa79fd98923d2a017a792082e2e1252d05e9e3

With the P6 context model, but with the fragment distribution of a multi-pass run, 2,077,088,916 bp of CLR data is generated for Chr1 the human reference genome human_g1k_v37_decoy.fasta. We selected fragment size of over 800 and number of passes between 10 and 15. The CCS reads are generated with the command:

- pbccs/bin/ccs –minPredictedAccuracy 0.0 output.bam *bam

The CCS reads from output.bam are extracted into a FASTQ file, which is mapped back to the full reference in human_g1k_v37_decoy.fasta with:

- bwa mem -x pacbio

The resulting alignment is evaluated against the truth bed file generated during the simulation with the following criteria:

(1) if the SAM record indicates unmapped, we classify as such
(2) if the SAM record overlaps with more than half of the truth locus, we classify a match, and the error fraction is estimated by NM/(length of aligned locus)
(3) otherwise, the record is classified as a mismatch

We then report an estimate of median accuracy as -10log10 (median error fraction). The result is listed in Table S4. The ratio of the number of CLR reads to CCS reads is around 14, as expected by the number of passes simulated. The result confirms that our simulated data can be processed by PBCCS2 for an improvement of Q 9 to Q 31, as expected from Ref. [8].

	Number of Reads	% Unmapped	% Mismapped	% Recovered	Median Accuracy (%)
CLR	1728699	1.29	5.49	93.22	Q$\sim$9
CCS	122672	0.00	0.38	99.62	Q$\sim$31

TABLE S 4. Comparison of simulated CLR reads and the corresponding CCS reads, as generated by PacBio's CCS2.

11. VarSim Evaluation

As a demonstration of VarSim+LongISLND evaluation of the PacBio pipeline, we evaluate variant calling for Chr21 of NA12878 using real and simulated data. The real P5C3 data is available at SRX627421[10]. Here, we note that SRX638310 is another NA12878 data set containing reads generated with pre-P5 chemistry. We managed to aligned reads from both SRX627421 and SRX638310, but we managed to run Quiver for the reads from only SRX627421 (not mixed with SRX638310), probably due to incompatible meta data stored in the H5 file. The simulated data is generated using the error profile from P5C3 CHM1 data as well as VarSim diploid simulation according to the NA12878 high-confidence call set. The lack of real ground truth of real data restricted us to evaluate the callsets over only high-confidence region, and the accuracy should be interpreted as an upper bound outside of the high-confidence region. Table S5 shows the germline variant calling result for Freebayes and Quiver with PacBio's continuous long reads (CLR). GATK Unified Genotyper failed to call any Ins/Del and Haplotype Caller failed make any calls. The run-time of the best-practice GATK pipeline at least doubles that of Freebayes (3 days for Chr21 on 12 physical cores) and is thus not reported. We see that the callers are challenged by the diploid nature of the simulation. Quiver can indeed call homozygous SNVs at high accuracy with 50X coverage; however, much higher coverage is required to call heterozygous SNVs. Freebayes actually has higher SNV accuracy at the same coverage, thanks to better heterozygous SNV calling performance. The two callers both have difficulties calling insertion and deletion, but Quiver manages to call at a higher rate. The low indel accuracy and low heterozygous SNV accuracy are known characteristics of diploid sequencing with PacBio CLR reads. For example, the recent publication of NA12878 PacBio+BioNano de novo assembly [10] reported that low number of SNV events and substantially high number of small Ins/Del when compared to Illumina call set.

We tested somatic mutation callers by simulating 100X coverage of normal genome and at least 100X of tumor genome generated by VarSim. Here, the CLR reads are generated using the P5 model, and the circular consensus reads (CCS) reads are generated using the P5 model extrapolated to 1% error. Table S6 shows that Vardict is clearly superior to PacBio's MinorVariant protocol, in terms of speed, accuracy, for the same coverage and read type.

12. Tables

Table S 5. F1 score of germline variant calling using real and simulated P5C3 data for NA12878. Evaluation is performed by VarSim over the high-confidence regions in Chr21 of NA12878.

Caller	Reads	Coverage		SNV			INS			DEL	
			ALL	HOM	HET	ALL	HOM	HET	ALL	HOM	HET
Quiver-diploid	SRX627421	∼30X	77.61	97.49	59.58	42.31	45.48	37.28	52.27	61.90	39.13
Quiver-diploid	LongISLND	30X	79.67	99.91	61.65	50.76	55.94	42.49	50.50	59.14	38.60
Quiver-diploid	LongISLND	50X	80.09	99.93	62.54	53.72	59.36	44.89	63.75	78.72	44.87
Quiver-diploid	LongISLND	100X	80.02	99.96	62.35	55.92	61.74	47.08	66.58	84.99	43.29
Quiver-diploid	LongISLND	200X	91.42	99.94	85.12	57.29	63.29	48.26	66.41	86.49	40.48
Freebayes	LongISLND	100X	84.99	99.80	73.04	48.33	64.08	8.04	35.89	42.42	26.43

Table S 6. F1 score of somatic variant calling with CLR and CCS reads emulating the CHM1 dataset.

	Read Type	Normal:Mixture Coverage	SNV	INS	DEL	Walltime
Vardict	CCS	100X:100X	0.99	0.50	0.50	4 hours
Vardict (no realignment)	CCS	100X:100X	0.99	0.13	0.29	2 minutes
MinorVariant	CCS	100X:100X	0.93	0	0	5 days
Vardict (no realignment)	CLR	100X:200X	0.91	0	0.14	10 minutes
Vardict (no realignment)	CLR	100X:100X	0.80	0	0	10 minutes
Vardict	CLR	100X:100X	0.55	0	0	20 hours
MinorVariant	CLR	100X:100X	0.01	0	0	10 days

References

[1] Konstantin Berlin et al. "Assembling large genomes with single-molecule sequencing and locality-sensitive hashing". In: *Nat Biotech* 33.6 (June 2015), pp. 623–630. URL: `http://dx.doi.org/10.1038/nbt.3238`.

[2] Mark J Chaisson and Glenn Tesler. "Mapping single molecule sequencing reads using basic local alignment with successive refinement (BLASR): application and theory". In: *BMC Bioinformatics* 13.1 (2012), p. 238. DOI: `10.1186/1471-2105-13-238`. URL: `http://dx.doi.org/10.1186/1471-2105-13-238`.

[3] Mark J. P. Chaisson et al. "Resolving the complexity of the human genome using single-molecule sequencing". In: *Nature* 517.7536 (Jan. 2015), pp. 608–611. URL: `http://dx.doi.org/10.1038/nature13907`.

[4] "http://www.ncbi.nlm.nih.gov/Traces/sra/?study=SRP044331". In: (2015).

[5] Nicholas J Loman, Joshua Quick, and Jared T Simpson. "A complete bacterial genome assembled de novo using only nanopore sequencing data". In: *Nat Meth* 12.8 (Aug. 2015), pp. 733–735. URL: `http://dx.doi.org/10.1038/nmeth.3444`.

[6] Pacific Biosciences. "BAM format specification for PacBio". In: (2016). URL: `https://github.com/PacificBiosciences/PacBioFileFormats/blob/3.0/BAM.rst`.

[7] Pacific Biosciences. "https://github.com/PacificBiosciences/DevNet/wiki/E.-coli-Bacterial-Assembly". In: (2015).

[8] Pacific Biosciences. "http://www.pacb.com/smrt-science/smrt-sequencing/accuracy/172869917286991728699". In: (2015).

[9] Pacific Biosciences. "SMRTAnalysis 2.3". In: (2013).

[10] Matthew Pendleton et al. "Assembly and diploid architecture of an individual human genome via single-molecule technologies". In: *Nat Meth* 12.8 (Aug. 2015), pp. 780–786. URL: `http://dx.doi.org/10.1038/nmeth.3454`.
